# Mechanical and thermodynamic properties of Aβ_42_, Aβ_40_, and α-synuclein fibrils: a coarse-grained method to complement experimental studies

**DOI:** 10.3762/bjnano.10.51

**Published:** 2019-02-19

**Authors:** Adolfo B Poma, Horacio V Guzman, Mai Suan Li, Panagiotis E Theodorakis

**Affiliations:** 1 Institute of Fundamental Technological Research, Polish Academy of Sciences, Pawińskiego 5B, 02-106 Warsaw, Poland; 2 Max Planck Institute for Polymer Research, Ackermannweg 10, 55128 Mainz, Germany; 3 Institute of Physics, Polish Academy of Sciences, Al. Lotników 32/46, 02-668 Warsaw, Poland; 4 Institute for Computational Science and Technology, Quang Trung Software City, Tan Chanh Hiep Ward, District 12, Ho Chi Minh City, Vietnam

**Keywords:** β-amyloid, atomic force microscopy, mechanical deformation, molecular simulation, proteins, α-synuclein

## Abstract

We perform molecular dynamics simulation on several relevant biological fibrils associated with neurodegenerative diseases such as Aβ_40_, Aβ_42_, and α-synuclein systems to obtain a molecular understanding and interpretation of nanomechanical characterization experiments. The computational method is versatile and addresses a new subarea within the mechanical characterization of heterogeneous soft materials. We investigate both the elastic and thermodynamic properties of the biological fibrils in order to substantiate experimental nanomechanical characterization techniques that are quickly developing and reaching dynamic imaging with video rate capabilities. The computational method qualitatively reproduces results of experiments with biological fibrils, validating its use in extrapolation to macroscopic material properties. Our computational techniques can be used for the co-design of new experiments aiming to unveil nanomechanical properties of biological fibrils from a point of view of molecular understanding. Our approach allows a comparison of diverse elastic properties based on different deformations , i.e., tensile (*Y*_L_), shear (*S*), and indentation (*Y*_T_) deformation. From our analysis, we find a significant elastic anisotropy between axial and transverse directions (i.e., *Y*_T_ *> Y*_L_) for all systems. Interestingly, our results indicate a higher mechanostability of Aβ_42_ fibrils compared to Aβ_40_, suggesting a significant correlation between mechanical stability and aggregation propensity (rate) in amyloid systems. That is, the higher the mechanical stability the faster the fibril formation. Finally, we find that α-synuclein fibrils are thermally less stable than β-amyloid fibrils. We anticipate that our molecular-level analysis of the mechanical response under different deformation conditions for the range of fibrils considered here will provide significant insights for the experimental observations.

## Introduction

All-atom molecular dynamics (MD) simulations have been employed to study the physical and chemical behaviour of the fundamental biomolecules of life (e.g., proteins [1], nucleic acids [2] and lipids [3]). Lipid membranes, viral capsids, and biological fibrils are common examples of large complexes that pose significant challenges for all-atom simulations. For example, the time scales of various biological processes are in the range from 10^−6^ to 10^−3^ s, and thus they are orders of magnitude larger than typical molecular motions (10^−15^ to 10^−12^ s) captured in all-atom MD. The length scales are similarly much smaller in all-atom simulations than it would be relevant for studying processes involving large conformational changes in large biological complexes. In the context of mechanical properties of various fibrils, for example, β-amyloids [4–5], cellulose [6] and collagen [7], all-atom models have been used to estimate the elastic moduli based on the response of the system, but mostly approximately. Still, molecular-level methods are necessary to understand the microscopic mechanisms of the mechanical response of biological fibrils. In this regard, coarse-grained (CG) models are suitable, because they remove several degrees of freedom of the system, which enables them to reach the experimental time and length scales that describe the relevant phenomena while maintaining a molecular-level description of the systems under consideration [8–11]. CG simulations are able to describe large structural changes in the context of fibril deformation, which would be otherwise impossible with all-atom models. In particular, the CG model can be used to infer the elastic parameter under ideal conditions, which is given by the Hertz model [12] and is valid for isotropic materials and as close as possible to the experimental conditions [13]. While other sophisticated “Hertz-based models” [14–15] aim to study the elastic properties of anisotropic materials with high symmetries, e.g., crystals, such descriptions are not suitable for softer materials such as biological fibrils or polymers. Although biological matter is an example of an anisotropic material, it is not expected to follow a priori a simple Hertzian relationship given by *F* ≈ *Y*_T_*h^3/2^* (with *Y*_T_ being the transversal Young modulus and *h* being the indentation depth). If it actually follows this relationship, then the elastic modulus can be easily obtained from the slope of the curve. This approach can be used to test the experimental estimation of an elastic property. Most importantly, the mechanism of deformation that gives rise to the linear response can be characterized in the CG simulations. From the experimental point of view, there is a long-standing discussion in the atomic force microscopy (AFM) community whether Hertzian mechanics is applicable to all soft-matter samples explored with AFM. One of the basic assumptions of the Hertz model is that the indented object is a half-space and made out of a homogeneous material. However, at the nanoscale it is intrinsically difficult to measure pure and homogeneous materials, or perfectly mixed materials, with some exceptional cases, such as highly oriented pyrolytic graphite (HOPG), silica, and other “clean” surfaces, which are, however, very far away from biological systems. Moreover, by considering the indenter as a sphere, the anisotropies in the deformed material can be screened, since the measured deformation depends on the contact area, which will be the arc region that forms in contact with the sphere. In considering other shapes for the cantilever tip, such as conical or flat punch, the impact of the anisotropy is expected to be much higher [16]. Nonetheless, to our knowledge the exact shape of the cantilever tip cannot be determined during experimental measurements. As a result, big discrepancies are found when comparing Young’s modulus values measured with macroscopic techniques and nanoscopic ones such as AFM. This is because a nanoscopic exploration of biological systems reaches molecular resolution and the measurements are in general very delicate due to the intrinsic properties of soft matter and the danger of damaging the samples [17]. As a matter of fact, the employed reference model to study the mechanical response of biological fibrils during AFM nanoindentation has been also the Hertz model. Hence, we also use it as a reference for comparing the indentation values we obtained to the experimental ones, although we remark that our molecular modeling can adapt further anisotropic mechanical models, envisioned within force microscopy techniques.

**Figure 1:**
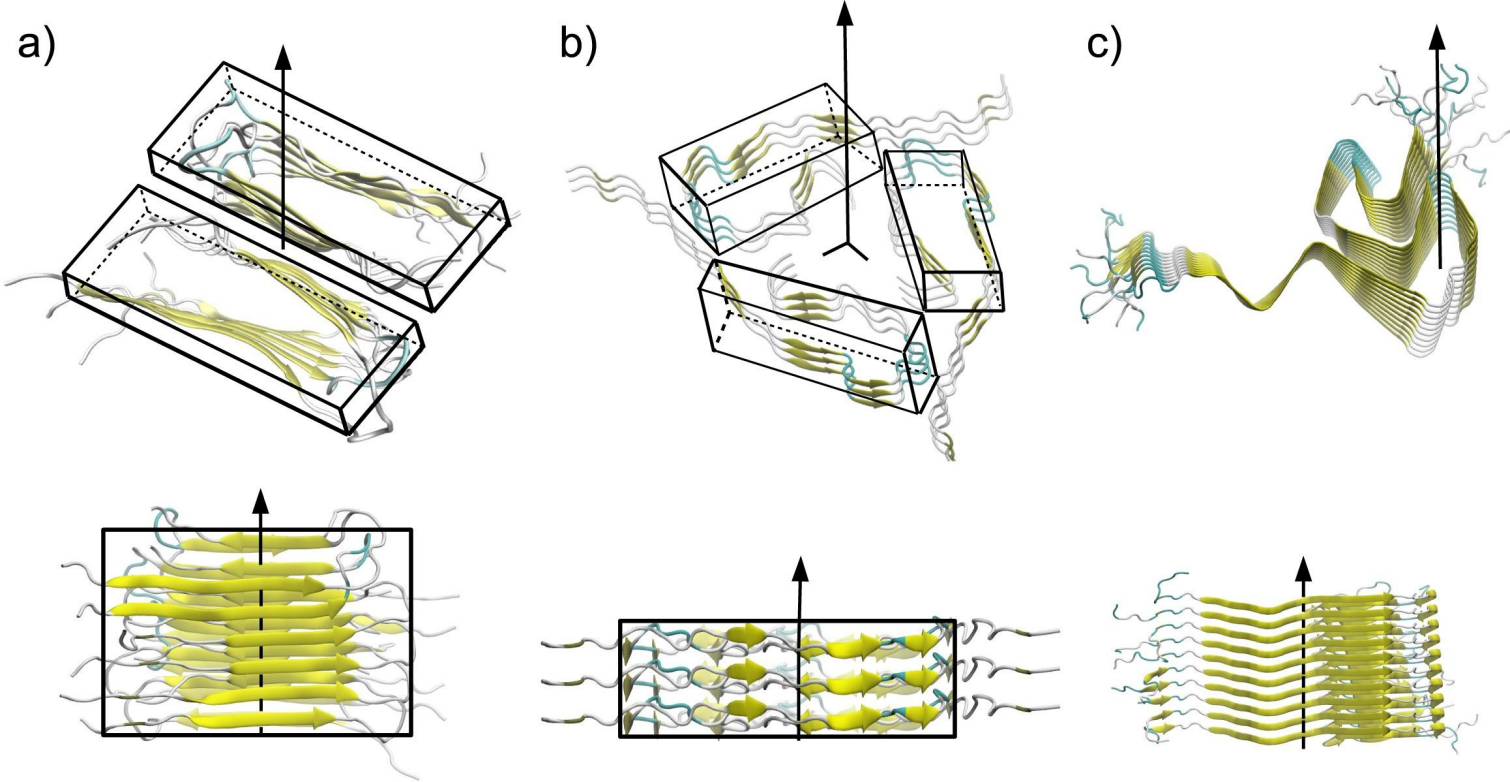
Snapshots illustrating some of the biological fibrils used in our simulation. The main axis of symmetry and the secondary structure for each chain are indicated. (a) Aβ_40_ (PDB ID: 2LMO) with two-fold symmetry. (b) Aβ_40_ fibril (PDB ID: 2M4J) with three-fold symmetry. (c) α-syn fibril (PDB ID: 2N0A) with no symmetry. Rectangular boxes depict the local symmetry.

Biological fibrils are well-known biomaterials of practical use. The related technological applications range from drug delivery [18] to structural scaffolds [19] in which the role of the fibril may be to immobilize small molecules such as enzymes [20]). The applications are motivated by the unique properties of fibrils, such as the spontaneous formation under certain conditions, the high mechanical stability (comparable to silk), and the ability to form ordered structures, albeit the monomeric units (proteins) of these fibrils are intrinsically disordered [21–22]. These are fundamental properties for applications in which the fragmentation of the material needs to be avoided, for example, syntheses, active processes (drug delivery) or responses to an external perturbation (change in temperature). To this end, the interplay between mechanical and thermodynamic properties will determine the overall behaviour of the fibrils, which depends on the arrangement of the individual amino-acid chains in these structures. Fibrils consisting of either 40-mer or 42-mer amyloid chains (it contains two additional hydrophobic amino acids) are particularly interesting. For example, Aβ_40_ typically assembles into two-fold or three-fold symmetries (see Figure 1), while the highest symmetry reported by experiments for Aβ_42_ fibrils is a two-fold symmetry, as in the case of α-synuclein (α-syn) fibrils [23–24]. Furthermore, the aggregation typically takes place 2.5-times faster in a solution of Aβ_42_ than in the case of Aβ_40_ [25–26]. Interestingly, the aggregation rate of fibril formation has been found to be highly correlated with the mechanical properties of the fibrils, namely, the mechanically more stable fibril is the one undergoing faster aggregation [27]. While experimental observations have been derived from a small set of samples, our CG simulations can be used to validate these observations and study a larger set of fibrils.

Typical length scales of biological fibrils are in the range from nanometers to micrometers. Hence, AFM that can be operated, for example, in static (contact) and dynamic modes, has been one of the main methods to study such systems [28–29]. On one hand, AFM in contact mode has been used to provoke the mechanical deformation of fibrils obtaining the Young’s modulus (here denoted as *Y*_T_) [30–32]. On the other hand, the experimental determination of the tensile Young’s modulus (*Y*_L_) is nontrivial at the nanoscale [33], due to the requirement of a different experimental setup, namely, the more involved sonification method [34]. Moreover, the experimental calculation of the shear modulus (*S*) can be realised by suspending the fibril between two beams and pressing the free part against the indenter, which gives rise to the fibril bending modulus (*Y*_b_) that depends on both *Y*_T_ and *S*.

Our CG strategy can be used to extract and compare elastic properties in a systematic way. This significant advantage of CG simulations has motivated the current study, which employs MD simulation of a structure-based CG model [35–38] to investigate one α-synuclein and five β-amyloid fibrils of known experimental structure related to specific neurodegenerative diseases. Our simulation sheds light on the mechanical and thermodynamic properties of these fibrils by providing the microscopic picture required to explain the relevant phenomena. We achieve this by applying different types of deformation (e.g., tension, shearing and indentation) and analysing the intermolecular contacts between amino acids. Our simulations reveal significant differences in the mechanical behaviour between Aβ_40_ and Aβ_42_ and α-syn fibrils. Moreover, we find that the α-syn fibril is thermally less stable than the β-amyloid fibrils.

In the next section, we present details about our methodology. Then, we present our results and analysis, and in the last section we summarise our conclusions.

## Materials and Methods

For our studies, we have chosen three different Aβ_40_ fibrils with the PDB IDs 2LMO [39], 2M4J [40] and 2MVX [41] and two Aβ_42_ with the PDB IDs 5OQV [42], and 2NAO [43]. The only available structure for α-syn is the one with PDB ID: 2N0A [44].

### The coarse-grained model

In our CG model, each amino acid is represented by a bead located at the *C*_α_-atom position. The potential energy between the beads reads:

[1]
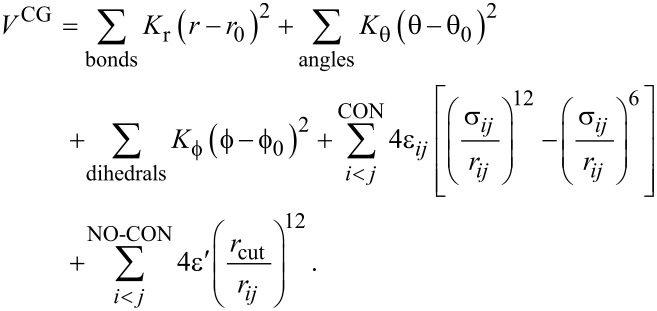


**Figure 2:**
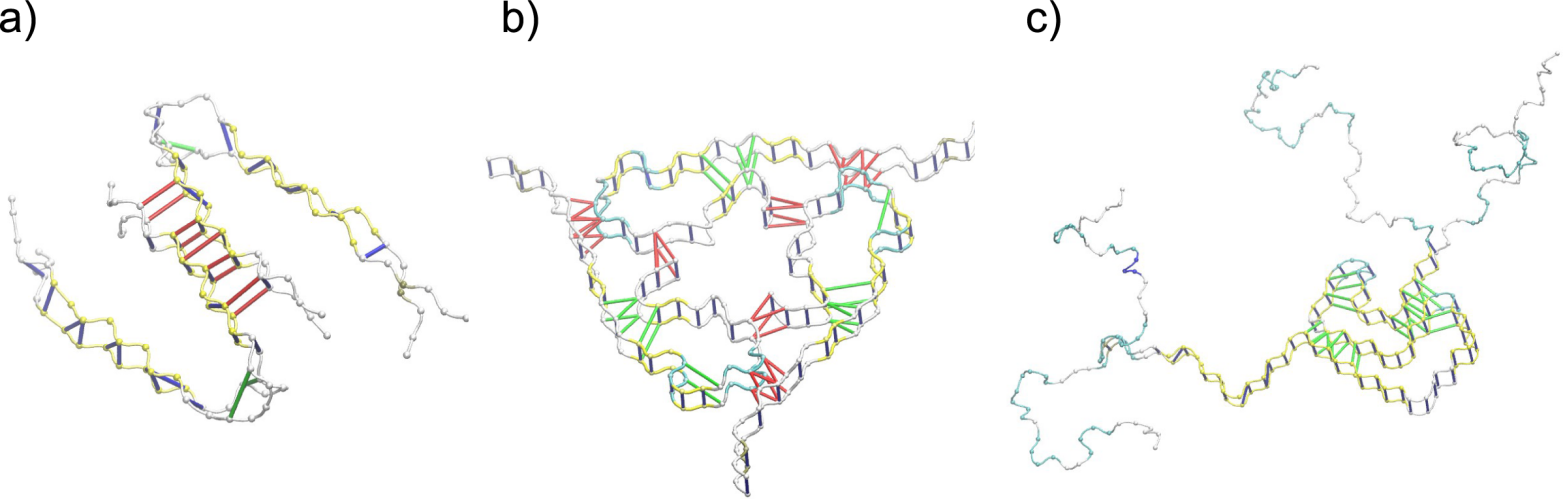
Coarse-grained representation of the biological fibrils presented in Figure 1. Illustrated are the three types of “native contact” interactions considered in our study: i) intrachain contacts (green), ii) interchain contacts (red) iii) intersheet contacts (blue).

The first three terms on the right hand side of Equation 1 correspond to the harmonic pseudo-bond, bond angle and dihedral potentials. The values of the elastic constants were derived from all-atom simulations [45] and are *K_r_* = 100 kcal/mol/Å^2^, *K*_θ_ = 45 kcal/mol/rad^2^ and 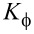 = 5.0 kcal/mol/rad^2^. The choice of equilibrium values *r*_0_, θ_0_, and 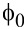 are based on two, three, and four α-C atoms, respectively, and are meant to favour the native geometry. The fourth term on the right-hand side of Equation 1 takes into account the non-bonded contact interactions, described by the Lennard-Jones potential. Here, we take ε*_ij_* to be uniform and equal to ε = 1.5 kcal/mol, which was also derived from all-atom simulations [45]. Our approach has shown very good agreement with experimental data on stretching [46–47] and nanoindentation of biological fibrils, such as virus capsids [35] and β-amyloids [36]. The strength of the repulsive non-native term, ε’, is set equal to ε. Our CG model takes into account native distances as in the case of a Gō-like model [37]. Hence, the native contacts are determined by the overlap criterion [48]. In practice, each heavy atom is assigned to a van der Waals radius, as proposed by Tsai and co-workers [49]. A sphere with the radius enlarged by a factor of 1.24 is built around the atom. If two amino acids have heavy atoms with overlapping spheres, then we consider a native contact between those two C_α_ atoms. In Figure 2, we show the CG representation for some biological fibrils as well as their native interactions. These native contacts represent hydrogen bonds (HB), and hydrophobic and ionic bridges. Moreover, we consider contacts between amino acids in individual chains with sequential distance |*i* − *j*| *>* 4. The parameters σ*_ij_* are given by *r_ij0_*/2*^(1/6)^*, where *r_ij0_* is the distance between two C_α_ atoms that form a native contact. The last term in Equation 1 simply describes the repulsion between non-native contacts. Here, we take *r*_cut_ = 4 Å. Moreover, our terminology for the “contacts” in this manuscript, is as follows: i) intrachain contacts are considered those within a single chain, ii) interchain contacts are between two chains in a side-by-side configuration and iii) the intersheet contacts are found along the symmetry axis (see Figure 2). Below, we provide details on the different types of mechanical deformation, i.e., tensile, shear, and indentation processes.

### Mechanical and thermodynamics characterization through a CG model

**Figure 3:**
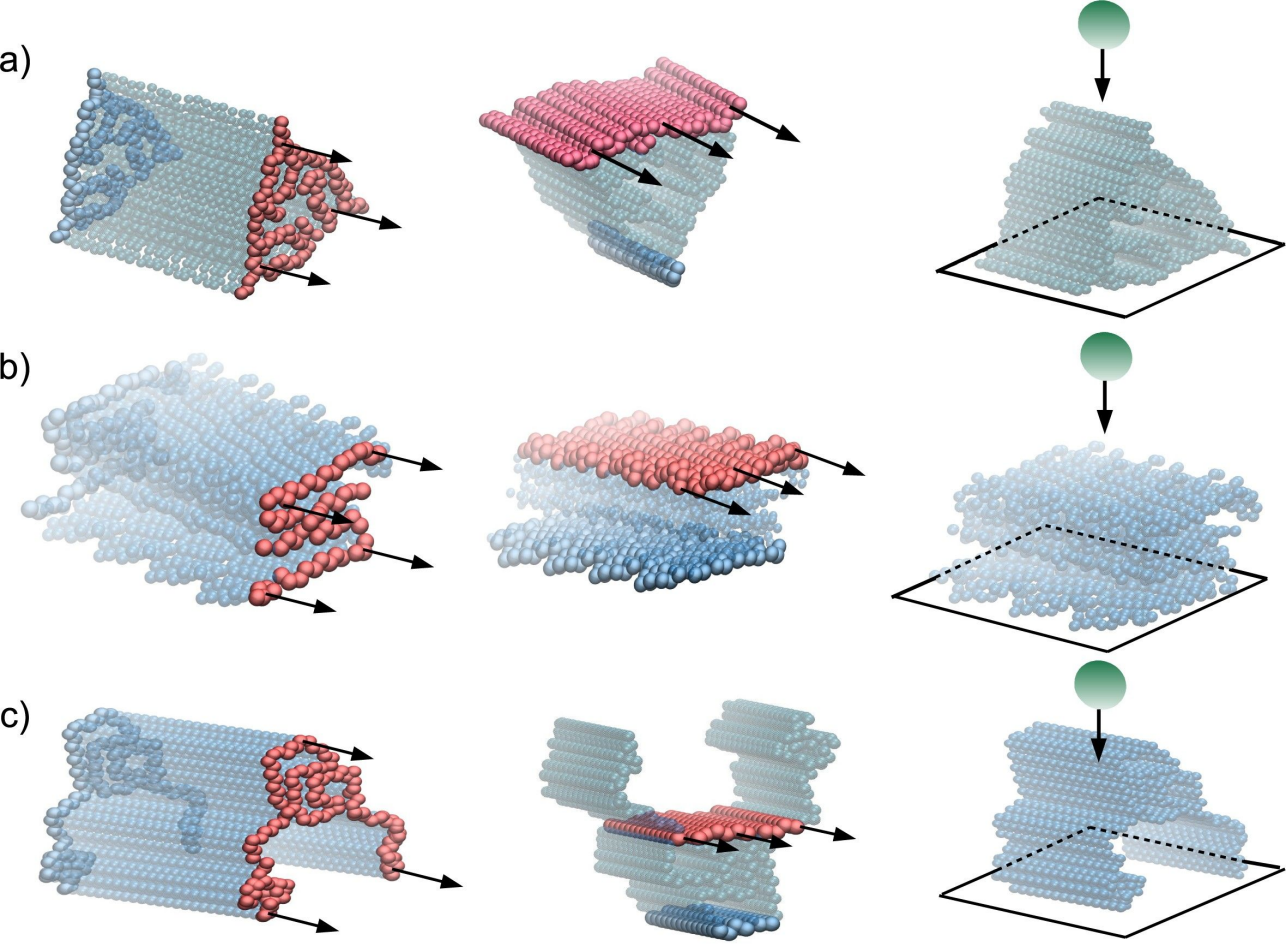
For the cases of Figure 1, we present schematically each deformation process. The left panels show tensile, the middle panels shearing, and the right panels indentation processes. The set of C_α_ atoms anchored in each processes are shown in blue, the ones which are moving at a speed *v*_pull_ are shown in red, and the indenter bead in green. Arrows indicate the direction of pulling. In the case of indentation, a potential *z*_0_^−10^ has been used to model the basis plane, where *z*_0_ is the distance between the plane and the CG beads. (a) Aβ_40_ (PDB ID: 2M4J), (b) Aβ_40_ (PDB ID: 2LMO) and (c) α-syn (PDB ID: 2NA0).

In our previous work [36], we have constructed a computational protocol for performing several types of mechanical deformation in silico (Figure 3). Such processes can be carried out at constant speed or force contact-modes. Here, we explore the former as it provides a dynamic picture of the whole process and it enables the characterisation of the mechanics during the early deformation stages. Moreover, we employ the CG simulation for the validation of the elastic theory. This is done by calculating the coefficient *n* in the indentation curves measuring the force as a function of *h^n^*. We found *n* = 3/2 in the linear regime, which corresponds to the Hertzian theory [12].

#### Tensile deformation

The experimental calculation of the stress–strain data at the nanoscale can be done by using optical tweezers (OT) [50], AFM base-force spectroscopy [51], or by the design of sophisticated microelectromechanical systems (MEMS) [52]. These techniques have been successfully used to predict the elastic properties of several biomolecules. However, OT are limited to applied loads below 0.1 nN and AFM has delicate calibration issues associated with a systematic deformation of samples of same length. In practice, all-atom simulation does not suffer from any of those drawbacks, but it cannot be used in biological systems. Instead, CG models are more suitable to achieve experimental length and time scales.

In practice, we set harmonic potentials to the furthest bottom and top particles of the protein. Then, we take values for the elastic constants equal to *k*_bottom_ = 100 kcal/mol/Å and *k*_top_ = 0.1 kcal/mol/Å for the top part of the fibril. The top part is moving with pulling speed equal to *v*_pull_ = 5 × 10^−5^ Å/ns. As a result of tensile deformation, the fibril stretches from a reference length (*L*_0_) to *L*, and the strain is given by 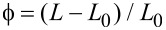. The stress is defined by the total force acting on the springs *k*_top_ divided by the cross-sectional area, *A*, of the sample. This area is calculated as follows [53]: For a given set of Cartesian points, it determines the smallest convex polygon containing all the given points. Then, we monitor the elementary area of this polygon during the simulation [54]. From the stress–strain plot one can derive the corresponding tensile Young’s modulus, *Y*_L_.

#### Shear deformation

The experimental techniques employed before for the determination of *Y*_L_ are not applicable for the calculation of the shear modulus (*S*) at the nanoscale. Hence, an improved version of the single three-point bending technique was developed for the calculation of *S* [55]. It combines a movement along the *z*-axis (perpendicular to the main fibril axis) with a continuous scanning motion along the main fibril axis. In this way, the slope d*F*/d*z* yields a better calculation of the bending modulus (*Y*_b_) and as a result a more accurate value of *S*. In comparison to its predecessor, this technique reduces the error in the value of *S* down to 12% in the case of collagen fibrils [55], but it still relies on the correct estimation of the fibril diameter. As above, the CG model helps to devise a protocol where simple shear planes can be applied on a set of atoms and the typical response allows, in a straightforward manner, for the calculation of *S*. In this case, we only couple the C_α_-atoms from the top (*k*_top_) and the bottom (*k*_bottom_) planes. The strain is defined by 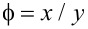, where *x* is the displacement of the top plane and *y* is the height of the fibril (see Figure 3). The shear stress is calculated as the total force acting on the top plane divided by the area of the plane (see in Table 1 the reference C_α_-atom used to define the top plane). From the stress–strain relation one can derive the corresponding shear Young’s modulus, *S*.

#### Indentation deformation

One of the empirical techniques used to estimate *Y*_T_ modulus is AFM nanoindentation. The wide range of applications of AFM technique span from biomolecules to single cells [31,56–57]. The AFM nanoindentation force–distance curves typically depend on the correct determination of the cantilever stiffness and only measurements of biological fibrils located at the centre of the fibril are considered. The former refers to the way that the indentation load is measured by the deflection of the AFM cantilever. The latter is an assumption of the semi-infinite half-space approximation. Once the AFM data is obtained, it requires interpretation by using a contact mechanics theory. There is no experiment at the nanoscale where the influence of the indenter could be neglected. Depending on the type of forces between the indenter and the biomaterial, we might describe the process by non-adhesive [12] or adhesive contact mechanics theories [58–59]. Here, we suggest our particle-based CG method as a tool for the modeling of the nanoindentation process. It is worth noting that we prevent any possible adhesion between the indenter and the fibril by placing a divergent interaction between the tip and the C_α_ atom, and hence other models [58,60] with such features are not considered. Moreover we chose the Young’s modulus of the indenter to be infinite and we define each system in the limit of the Hertzian theory [12]. The indenter is a sphere with a radius of curvature *R*_ind_ that moves towards the fibril with a speed *v*_ind_. Then, the penetration or indentation depth (*h*) is measured from the first tip–particle interaction (or contact) and the associated indentation force (*F*) is calculated until the indenter stops being in contact with the fibril. From Hertz’s relation, it follows that


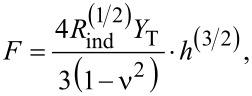


where ν is the Poisson coefficient, in this case equal to 0.5. This value corresponds to a homogeneous deformation in the *xy*-plane. From Hertz’s equation, we derive the transverse Young modulus, *Y*_T_, in the linear regime of the *F*–*h* curve.

#### Thermodynamic characterization

The study of the thermal stability in the case of Aβ fibrils faces serious difficulties, stemming from the requirement for controlled in vitro preparation of samples with well-ordered Aβ_40_ or Aβ_42_ fibrils. In this regard, our CG simulation is an ideal protocol as it enables the calculation of the melting temperatures for well-ordered Aβ fibrils. To assess the thermal stability of the fibril, we compute the probability of finding the protein in the native state, *P*_0_, as a function of the temperature *T*. We define the temperature of thermodynamic stability, *T*_f_ (folding temperature in our model), for the case *P*_0_ = 1/2. To study the thermodynamic properties of the biological fibrils, we carried out overdamped Langevin dynamics simulations. The simulations were performed for 35 different temperatures, *T*, which were uniformly distributed in the range from 0.1ε/*k*_B_ to 0.7ε/*k*_B_. Each simulation was 10^4^τ long after running the systems for 10^3^τ in order to reach equilibrium. In our studies, the unit of time, τ, is of the order of 1 ns. For this range of temperatures and time scales, we did not observe any dissociation or unfolding events for the fibrils. The deviation of the fibril structure from its native state was computed by means of the root mean square deviation (RMSD), which is defined as follows:

[2]
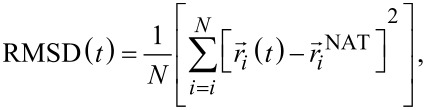


where 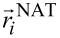 denotes the positions of the C_α_ atoms in the native state and 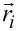 are the positions of the C_α_ atoms at time *t* after superimposing the native structure. After equilibration, RMSD fluctuates around an average value, 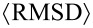, which is a function of the temperature *T*. In our case, the observed deviations from the native state in terms of RMSD are small at room temperature.

## Results and Discussion

### Tensile deformation

**Figure 4:**
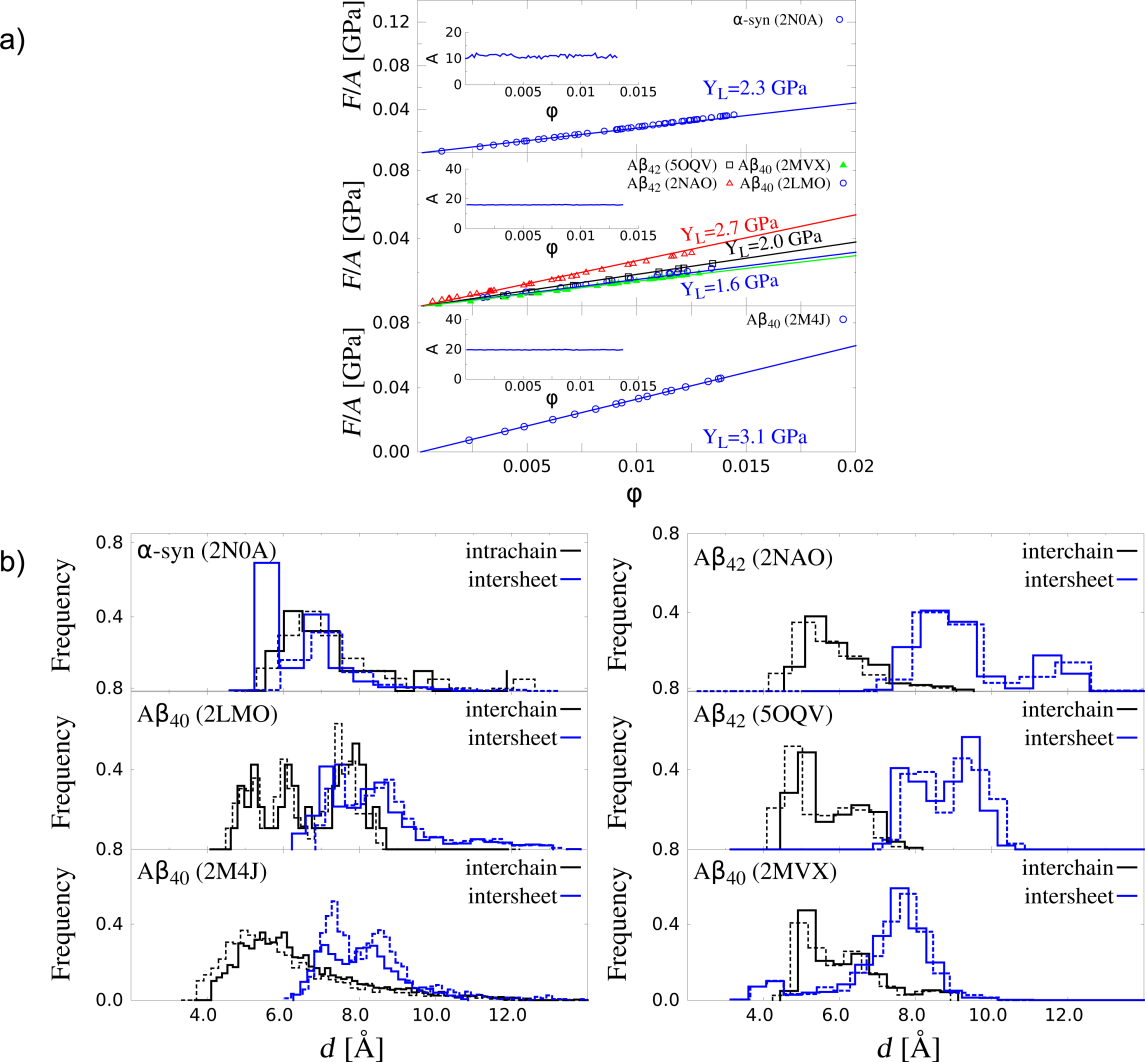
Results of tensile deformation. (a) Stress–strain curves of α-synuclein, three Aβ_40_ and two Aβ_42_ fibrils. Circles correspond to *v* = 0.0005 Å/τ. The error bars are the same as the symbol size and they are based on 50 independent simulations for each structure. The insets show the corresponding cross-section areas in nm^2^ for the corresponding pulling speed. (b) Distributions of HB lengths for 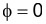 (solid lines) and for a finite strain 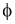 corresponding to the end of the linear regime (dashed lines): for α-syn the final 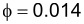, while 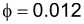 for Aβ amyloids.

Our results for tensile deformation for all studied cases are illustrated in Figure 4. The initial length (*L*_0_) is measured after an equilibration of 100 τ. The cross-section area (*A*) for each system is monitored during the simulation and is shown as a function of the strain in the insets of Figure 4. The deviations are small compared to the mean value, especially in the case of β-amyloid fibrils. Hence, we calculated the stress using the average value of *A*. The values of the cross-section areas and the initial length for each fibril are listed in Table 1. The theoretical values of *Y*_L_ have been obtained for *v*_pull_ = 0.0005 Å/τ are listed in Table 2, next to the experimental values for the sake of comparison. In our studies, the deformation is carried out along the main axis of symmetry (see Figure 1) for Aβ and α-syn fibrils. We find that the type of Aβ fibrils plays a more important role in the mechanical properties than the symmetry of each fibril. This becomes apparent by comparing the values of the tensile Young’s modulus of Aβ_40_ and Aβ_42_. Our discussion is based on the average values of *Y*_L_. In the case of Aβ_40_, we obtain *Y*_L_ = 2.1 GPa, while for Aβ_42_ this value is 2.4 GPa. The value *Y*_L_ = 2.3 GPa in the case of α-syn seems to be half way between the values for Aβ_40_ and Aβ_42_ fibrils. Moreover, our *Y*_L_ values are close to the experimental values of collagen fibrils equal to 1.9–3.4 GPa [61]. The bottom panels in Figure 4 illustrate the length distributions for the “native contacts” (intrachain, interchain, and intersheet) as defined in our CG model (Figure 2). We observe that the intersheet contacts become stretched, an effect that is independent of the system in terms of symmetry or type of individual chains (Aβ_40_ and Aβ_42_). In contrast, the interchain contacts, which keep together Aβ chains in the cross-section area, reduce their length. Moreover, in the case of α-syn there are no interchain contacts given that there is only one chain at the cross-section. In this case, only the intrachain contacts stretch during tensile deformation. A similar mechanism is found in Aβ fibrils (data not shown), which is consistent with the expectation of a constant cross-section area in the linear regime used to calculate the Young’s modulus.

**Table 1:** List of geometric parameters of the fibril structures used to determine the *Y*_L_, *Y*_T_, and *S*. The last line of each fibril entry gives the protein segment used to define the shear plane as illustrated in Figure 3.

Aβ_40_	2LMO	2MJ4	2MVX

initial length, *L*_0_ [nm]	41.10 ± 0.23	42.21 ± 0.34	29.10 ± 0.31
cross-section area, *A* [nm^2^]	16.02 ± 0.20	21.11 ± 0.33	19.20 ± 0.41
shear plane area, *A* [nm^2^]	160.01 ± 0.11	170.20 ± 0.41	131.00 ± 0.41
residue-id involved in shear plane	Gln15–Asp23	Asp1–Ala23,Asp1’	Gly9–Gly24
Aβ_42_	5OQV	2NAO	
initial length, *L*_0_ [nm]	29.30 ± 0.23	29.10 ± 0.31	
cross-section area, *A* [nm^2^]	17.30 ± 0.11	14.20 ± 0.34	
shear plane area, *A* [nm^2^]	123.00 ± 0.10	140.10 ± 0.11	
residue-id involved in shear plane	Tyr10–Asp23	Asp1–Asp7,Glu22–Gly25	
α-syn	2N0A		
initial length, *L*_0_ [nm]	45.20 ± 0.31		
cross-section area, *A* [nm^2^]	11.30 ± 0.41		
shear plane area, *A* [nm^2^]	160.00 ± 0.24		
residue-id involved in shear plane	Lys45–Glu105		

**Table 2:** The elastic moduli in GPa for the Aβ_40_, Aβ_42_ and α-syn from experiment and our CG model. The structural symmetry of β-amyloid (if specified in the literature) is given next to the PDB entries. The experimental results regarding indentation for Aβ_42_ and α-syn have been taken from [30]. The experimental values for the shear modulus (*S*) for β-amyloids have been taken from [62], while the experimental values of *S* and *Y*_L_ for α-syn are currently unknown.

tensile (*Y*_L_)/PDB ID	symmetry	Aβ_40_	Aβ_42_	α-syn

2LMO	2-fold	1.6 ± 0.1		
2MJ4	3-fold	3.1 ± 0.1		
2MVX	2-fold	1.5 ± 0.1		
5OQV	2-fold		2.0 ± 0.2	
2NAO	2-fold		2.7 ± 0.2	
2N0A	—			2.3 ± 0.2
Exp	—	—	—	—
shear (*S*)/PDB ID				
2LMO	2-fold	0.6 ± 0.3		
2MJ4	3-fold	1.2 ± 0.2		
2MVX	2-fold	0.4 ± 0.1		
5OQV	2-fold		1.3 ± 0.2	
2NAO	2-fold		1.8 ± 0.1	
2N0A	—			0.7 ± 0.2
Exp	—	0.1 ± 0.02	—	—
indentation (*Y*_T_)/PDB ID				
2LMO	2-fold	3.0 ± 0.1		
2MJ4	3-fold	6.0 ± 0.2		
2MVX	2-fold	5.0 ± 0.1		
5OQV	2-fold		7.0 ± 0.3	
2NAO	2-fold		16.0 ± 0.4	
2N0A	—			13.0 ± 0.1
Exp	—	—	3.2 ± 0.8	2.2 ± 0.6

### Shearing deformation

**Figure 5:**
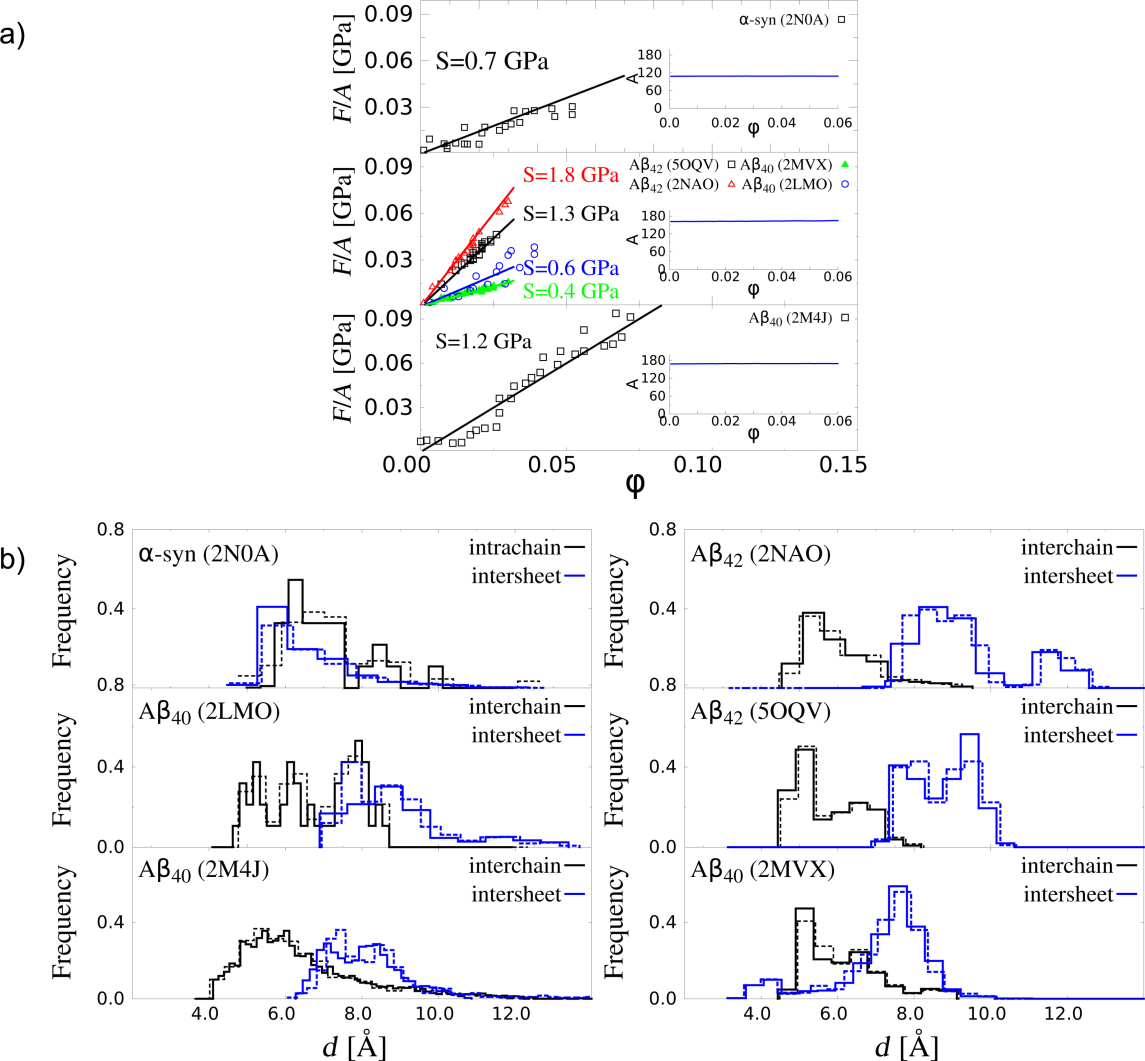
Results for shear deformation. (a) Stress–strain curves of α-syn and three Aβ_40_ and two Aβ_42_ fibrils. Circles refer to *v* = 0.0005 Å/τ. The error bars are the same as the symbol size and they are based on 50 independent simulations for each structure. The inset shows the corresponding cross-section area in nm^2^. (b) Distributions of the HB lengths for 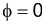 (solid lines) and for a finite 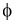 corresponding to the end of the linear regime (dashed lines), which is 0.04 for α-syn and 0.025 for Aβ amyloids. In the case of Aβ_40_ with PDB ID: 2M4J has been calculated at strain 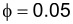.

Our results for all systems are presented in Figure 5. The shear deformation for Aβ and α-syn fibrils takes place along the same direction as the tensile deformation (see Figure 3). The initial values of the top-plane areas for each fibril are listed in Table 1. The insets in the left panels of Figure 5 demonstrate that the area *A* does not change when shear is applied. The values of shear modulus (*S*) computed for *v*_pull_ = 0.0005 Å/τ are listed in Table 2. In our studies, these values show a large dependence on the type of Aβ fibril. We find that *S* for Aβ_42_ is about 1.6 GPa, while for Aβ_40_ it is equal to 0.7 GPA. The 2.3-fold increase supports the picture that the Aβ_42_ fibril is mechanically more stable than the Aβ_40_[27]. The *S* value for α-synuclein is comparable to the Aβ_40_. No experimental data of *S* for α-synuclein fibril has been reported, but it is expected to be in the range of 1.4–300 MPa. Both limits are typical of microtubules [63] and collagen [55] systems, which are assemblies of proteins. Discrepancies between our computational studies and experimental results are expected. One of the sources of divergence is associated with the crystal-like regions that are present in the biological fibrils during each deformation in silico. The initial structure of fibrils is very close to the minimum free energy state (native). Here, the number of hydrogen bonds that participate in the deformation as a whole is larger as reported by all-atom simulations [4–5]. In contrast, during in vitro self-assembly of neurodegenerative fibrils the fibrilization process is dominated by extended regions of amorphous aggregates. Such regions will induce the overall softening of the fibril and therefore a drop in the elastic modulus.

Figure 5b shows the distributions of the characteristic native distances (see Figure 2 for their definition). For β-amyloid and α-synuclein fibrils, the intersheet contacts become slightly stretched, but the distances in the interchain contacts within each sheet are not affected in the case of amyloids. The same analogy can be seen for the intrachain contacts in α-syn fibril. This effect helps the system to keep constant the thickness of the fibril, a condition for the calculation of shear modulus in the linear regime.

### Indentation deformation

**Figure 6:**
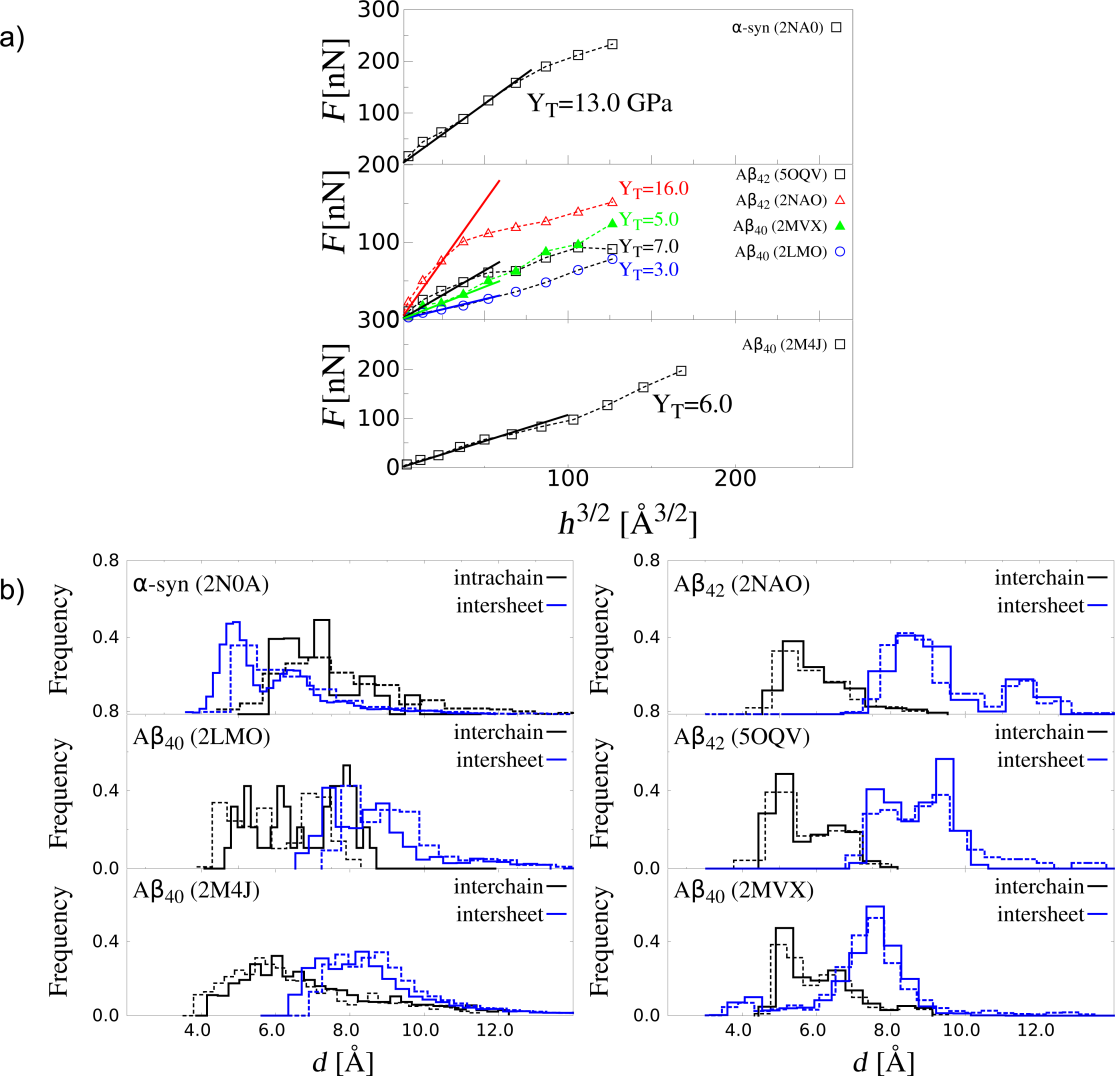
Nanoindentation deformation results for different biological fibrils. (a) Force as a function of the indentation depth (*h*) for α-syn, three Aβ_40_, and two Aβ_42_ fibrils. Square symbols refer to *v*_ind_ = 0.005 Å/τ and *R*_ind_ = 10 nm. The error bars are the same as the symbol size and they are based on 50 independent simulations for each system. The distributions are calculated for *h* = 0 (solid line) and *h* = 20 AA in the case of α-syn fibrils and Aβ fibrils (dashed lines). Only in the case of Aβ_42_ with PDB ID: 2NAO the value *h* = 9 Å was considered.

Our results for all systems are presented in Figure 6. The indentation deformation for Aβ and α-syn fibrils takes place in the normal direction to the plane *z* = 0 and at the position *L* = 0.5*L*_0_ (see Figure 3). The initial values of the fibril length for each fibril are listed in Table 1. The values of transversal Young’s modulus (*Y*_T_) computed for *v*_pull_ = 0.005 Å/τ are listed in Table 2. In the case of Aβ our results show a large dependency on the type of Aβ fibril. We determine that *Y*_T_ for Aβ_42_ is about 12 GPa, while for Aβ_40_ it is equal to 5 GPA. The 2.5-fold increase supports the picture that the Aβ_42_ fibril is mechanically more stable than Aβ_40_ [27]. Since Aβ_42_ aggregates faster than Aβ_40_ [64] our findings support the correlation between mechanical stability and aggregation propensity as in [27]. The *Y*_T_ value for α-synuclein is comparable to that of Aβ_42_. The experimental data on *Y*_T_ for α-syn fibril has been reported [30] and it is by a factor of two smaller than that of Aβ_40_. Such difference is attributed to an uncontrollable growth of amorphous aggregates during fibrillization that makes the fibril softer. But it is worth mentioning that our theoretical values can be considered as an upper bound in the case of highly ordered fibrils. Moreover, the same result has been observed in all-atom simulation studies [5].

Figure 6b shows the distributions of the characteristic native distances (see Figure 2 for their definition). For Aβ and α-syn fibrils, the intersheet contacts become stretched, but the distances in the interchain contacts within each sheet are shortened in the case of βA fibrils. Analogous effect can be seen for the intrachain contacts in α-syn fibril.

### Thermodynamic characterization of fibrils

**Figure 7:**
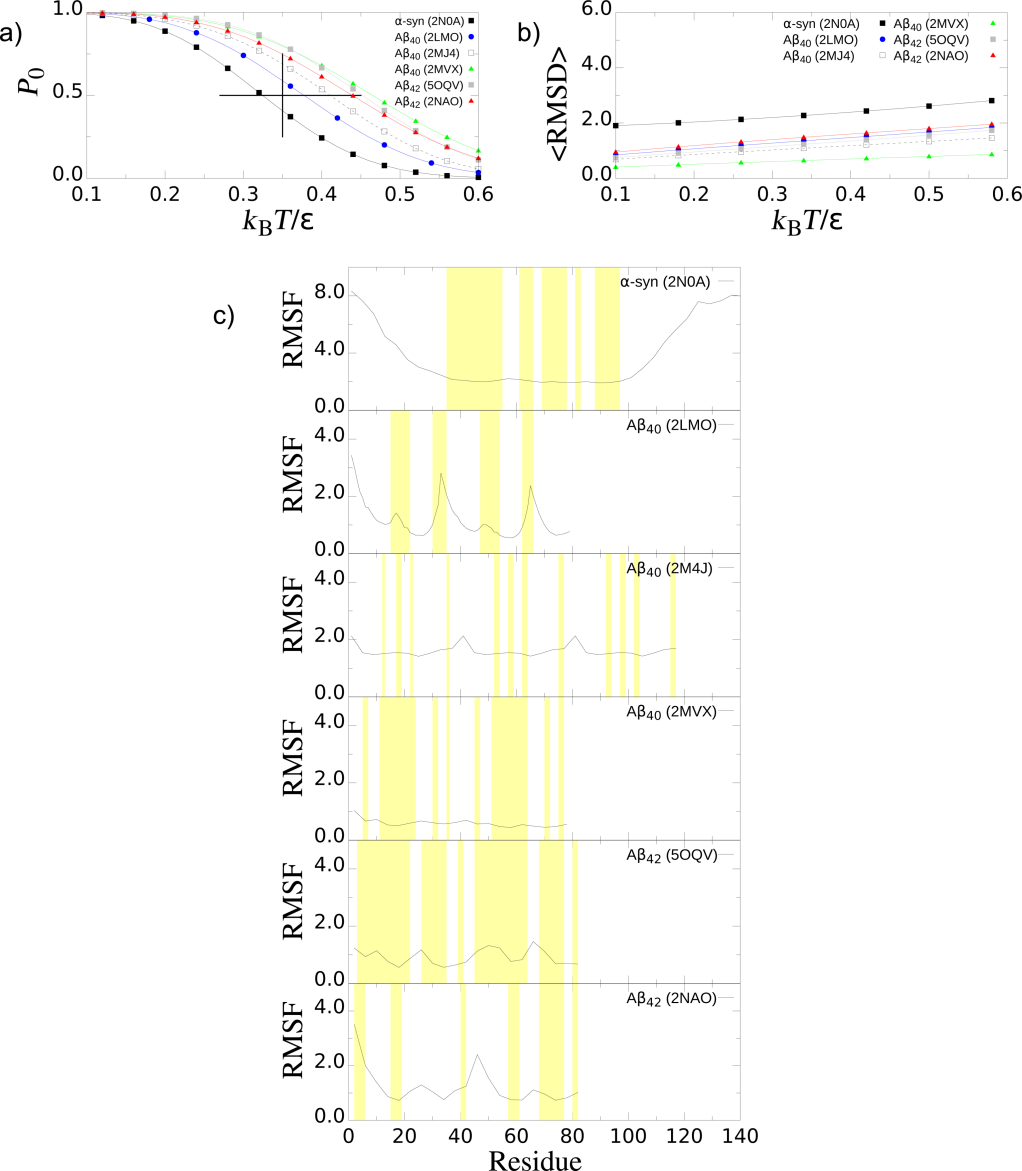
Thermodynamic properties of biological fibrils. (a) Probability of finding the fibrils in the native “fibril” state , *P*_0_, as a function of the temperature. The vertical line indicates the room temperature equal to 0.35ε/*k*_B_ and the horizontal line the range of temperatures that offer thermodynamic stability in our model. (b) RMSD of the fibrils. (c) Root-mean-square-fluctuation (RMSF) at room temperature. The β-strand segments in each system are highlighted in yellow.

Our results regarding the effect of the temperature for each fibril structure are presented in Figure 7. We first study *P*_0_ for all fibrils as a function of the temperature. Figure 7a shows that the probability *P*_0_ of finding the fibrils in the native state is larger for Aβ_40_ and Aβ_42_ than for α-syn at any given temperature. This result is in agreement with a differential calorimetry experiment where it is observed that *T*_m_ of β-amyloid fibrils is larger than that of α-syn fibrils [65–66]. In the case of the single fibril, Aβ_40_ (PDB ID: 2MVX) with two-fold symmetry, it is the most stable at higher temperatures (thermophilic character) among the other two-fold and three-fold β-amyloids. The calibration of our room temperature is 0.35ε/*k*_B_. In particular, the folding temperature (*T*_f_) defined in our CG model at *P*_0_ equal to 0.5 gives *T*_f_ equal to 0.38, 0.42, 0.44, 0.46, and 0.48 in units of ε/*k*_B_ for the amyloids with the PDB entries 2LMO, 2MJ4, 2NAO, 5OQV, and 2MVX, respectively. With our calibration of ε, the difference between the most (PDB ID: 2MVX) and least (PDB ID: 2LMO) thermophilic fibrils is of the order of 85 °C. Our results indicate that the α-syn fibril is thermally less stable than the Aβ system and this behaviour seems to be intrinsically associated with the extended disordered N-terminus and C-terminus domains. In our model, for α-syn we have determined that *T*_f_ is 0.33ε/*k*_B_. The difference in temperature with respect to Aβ with PDB IDs 2LMO and 2MVX is 43 °C and 128 °C, respectively. This implies a higher thermodynamic stability of the Aβ systems in comparison with α-syn, which may explain the easier formation of Aβ fibrils over α-syn fibrils. Figure 7b shows that 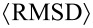 is larger in the case of α-syn than in the case of Aβ fibrils, at any given *T*. In addition, Figure 7c presents the RMSF results for all fibrils. We observe that the disordered domains (N- and C-terminus) in α-syn are very flexible in comparison with Aβ fibrils.

## Conclusion

We have carried out molecular dynamics simulations to study the elastic properties of two families of biological fibrils, namely, the β-amyloid and α-synuclein. The elastic properties of this study are the tensile, shear, and indentation deformations. Overall, our results are in agreement with the corresponding experimental values that could be obtained from the literature. Moreover, our method is sensitive to variations in the chain length and the symmetry of the β-amyloid fibril. Our results indicate a higher mechanostability in the case of βA_42_ fibrils than in the case of Aβ_40_, namely, 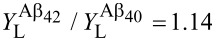, 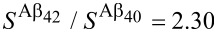, and 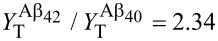. This result is consistent with the results obtained by means of the rupture force [27]. Most importantly, given that the aggregation rate depends on the mechanical stability of the fibrils [27] our study could provide also hints for the self-assembly of β-amyloid and α-synuclein chains. Our results also indicate an elastic anisotropy namely, *Y*_T_ *> Y*_L_, for all systems. In the case of α-syn fibrils this difference between *Y*_T_ and *Y*_L_, is almost one order of magnitude. In contrast, in the case of β-amyloid fibrils the anisotropy is considerably smaller.

We find that this effect is due to the deformation of the hydrophobic core (segments 61–95). We have also confirmed that the large anisotropy in the case of α-syn neither depends on the N-terminus nor the C-terminus domains. Although the mechanical properties indicate some similarity between α-syn and Aβ fibrils, thermodynamic properties reveal differences. That is, β-amyloid fibrils are thermally more stable than α-syn fibrils. β-Amyloid fibrils are, in general, more stable at higher temperatures than at room temperature, while the opposite is the case for α-syn fibrils. In this regard, our method can be used to explore systematically the temperature dependence of the mechanical properties (thermoelasticity) in biological fibrils at experimental length and time scales.
